# Supplementary material for: Learning curve and surgical outcome of robotic assisted colorectal surgery with ERAS program
Source: Sci Rep. 2022 Nov 29;12:20566. doi: 10.1038/s41598-022-24665-w (PMC9709162; doi:10.1038/s41598-022-24665-w)
Supplement: Supplementary file 2 — Supplementary Information 2. [file 41598_2022_24665_MOESM2_ESM.zip › RAL Raw data-Ñ[▒K/102/OP2_ñw╝╨░O▒Kñσ.pdf]

正式摘要(電子簽章：000000221715953)

W83 / W83

臺中榮民總醫院

Operation Note

[redacted]

- 手術日期：2021/07/29
- 手術醫師：VS 李建儀，VS 胡如娟
- Anesthesia : GE
- Position : Lateral-Lt
- Aseptic agents : Soap , Betadine Alcohol

Diagnosis

- Pre-operative diagnosis : Rt U/3 ureter stone with severe hydronephrosis
- Post-operative diagnosis : Ditto. s/p robotic assisted Rt ureterolithotomy + 6*24 DJ insertion

Surgical Indication

- resolve obstruction uropathy

Operation

- robotic assisted Rt ureterolithotomy + 6*24 DJ insertion

Finding

- 1. Rt U/3 ureter 2cm stone with severe dilation above obstruction level
  2. 6*24 DJ was inserted after stone removal

Procedure

- 1.After robotic LAR, the patient was turn to left decutitus position. Trocars for robotic LAR were used and a RUQ 8mm trocar was added for robotic Rt hand (scissor). Fenestrate was used as left hand device.
  2.A colon was taken down first. Kocker maneuver was also performed. The ureter was identified above bification level. After looping ureter, tracining the ureter to the stone impaction level.
  3.The ureter was incised longituidinally. The stone was removed.
  4.6*24 DJ was placed through assistance port via hydrophilic guidewire open method.
  5.4-O Vicryl interrupted sutures were applied transversely to prevent further ureter stricture.
  6.10mm penrose was placed over suture site.
  7.The wound was closed layer by layer. The process was smooth.
- Estimated blood loss: minimum
- 器械數量: 正確
- 紗布數量: 正確

Image

- PAINT
  - abdomen

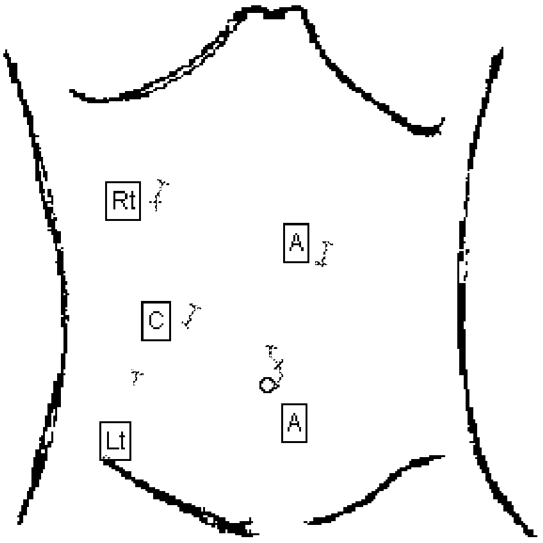

編輯主治 2274C胡如娟

正式摘要(電子簽章：000000221715953)

W83 / W83

doctorRank:B
